# Convolutional Recurrent Neural Network for Dynamic Functional MRI Analysis and Brain Disease Identification

**DOI:** 10.3389/fnins.2022.933660

**Published:** 2022-07-06

**Authors:** Kai Lin, Biao Jie, Peng Dong, Xintao Ding, Weixin Bian, Mingxia Liu

**Affiliations:** ^1^School of Computer and Information, Anhui Normal University, Wuhu, China; ^2^Department of Radiology and Biomedical Research Imaging Center (BRIC), University of North Carolina at Chapel Hill, Chapel Hill, NC, United States

**Keywords:** dynamic functional connectivity, sequential information, Alzheimer's disease, classification, fMRI

## Abstract

Dynamic functional connectivity (dFC) networks derived from resting-state functional magnetic resonance imaging (rs-fMRI) help us understand fundamental dynamic characteristics of human brains, thereby providing an efficient solution for automated identification of brain diseases, such as Alzheimer's disease (AD) and its prodromal stage. Existing studies have applied deep learning methods to dFC network analysis and achieved good performance compared with traditional machine learning methods. However, they seldom take advantage of sequential information conveyed in dFC networks that could be informative to improve the diagnosis performance. In this paper, we propose a convolutional recurrent neural network (CRNN) for automated brain disease classification with rs-fMRI data. Specifically, we first construct dFC networks from rs-fMRI data using a sliding window strategy. Then, we employ three convolutional layers and long short-term memory (LSTM) layer to extract high-level features of dFC networks and also preserve the sequential information of extracted features, followed by three fully connected layers for brain disease classification. Experimental results on 174 subjects with 563 rs-fMRI scans from the Alzheimer's Disease Neuroimaging Initiative (ADNI) demonstrate the effectiveness of our proposed method in binary and multi-category classification tasks.

## 1. Introduction

Alzheimer's disease (AD) is the most common neurodegenerative disease in the elderly, accounting for about two-thirds of all dementia cases, which can cause irreversible loss of brain neurons (Nussbaum and Ellis, 2003). The clinical manifestations are progressive impairment of memory, insight, and judgment, as well as obstacles to the orientation of the surrounding environment and language obstacles (Tarawneh and Holtzman, 2012). The cost of long-term care for patients with AD may bring a heavy economic burden to the family and society (Association, 2016). Mild cognitive impairment (MCI), the prodromal stage of AD, may develop into clinical AD at a fairly rapid rate (Petersen et al., 2001). Therefore, accurate diagnosis of MCI and AD is of great significance for early treatment and prevention of disease progression.

Resting-state functional magnetic resonance imaging (rs-fMRI), which measures low-frequency fluctuations in blood oxygen level dependent (BOLD) signals, provides a non-invasive solution for studying the functional structure of the brain (Lee et al., 2013). Functional connectivity (FC) networks derived from rs-fMRI data help describe the temporal dependency of neuronal activation patterns between brain regions (van den Heuvel and Hulshoff Pol, 2010), and have been applied to the automated diagnosis of brain diseases, such as AD and MCI (Chen et al., 2011; Wee et al., 2012), schizophrenia (Shen et al., 2010), and autism spectrum disorder (Wang et al., 2019c). The existing studies usually implicitly assume that the FC of the human brain is *stationary* during fMRI recording (Sporns, 2011), thus ignoring the temporal dynamics of the brain network. Many studies have shown that, even in the resting state, FC also exhibits significant temporal dynamic changes (Hutchison et al., 2013; Zhang et al., 2016). Increasing evidence suggests that the dynamics of FC networks may be related to cognitive states (Chang et al., 2013; Thompson et al., 2013), and assessing the dynamics of FC networks is critical for better understanding the pathology of brain diseases (Zhang et al., 2016). Several recent studies have resorted to dynamic FC (dFC) network to characterize the temporal changes of FC between brain regions (Hutchison et al., 2013) and investigated the association of changes in dFC networks with brain diseases (Jones et al., 2012). Also, studies have applied *dynamic* FC networks to brain disease classification (Sakoglu et al., 2009; Wee et al., 2016), and achieved better performance compared with those that use traditional stationary FC networks.

Existing studies on dFC network analysis can be roughly categorized into two categories: (1) traditional machine learning methods, and (2) deep learning methods. The first category typically extracts low-level network measures (i.e., clustering coefficients) as rs-fMRI features and trains a learning model (i.e., support vector machine, SVM) for classification (Wee et al., 2016). For example, Jie et al. (2018) extract and integrate temporal and spatial variability of dFC networks for MCI classification. These methods usually rely on handcrafted feature representations for classification/prediction models, thereby producing sub-optimal classification performance. In contrast, deep learning methods usually learn features of dFC networks in a data-driven manner for brain network analysis (Kawahara et al., 2016). For example, several studies have applied convolutional neural network (CNN) to the automated diagnosis of brain diseases (Wang et al., 2019b; Jie et al., 2020). Compared with the traditional machine learning methods, deep learning methods could automatically learn data-driven features from dFC networks and provide a unified framework for feature learning and classification/prediction, thus achieving good classification performance. However, these methods usually ignore the sequential information of dFC networks that could be used to further improve the performance.

**Figure 1:**
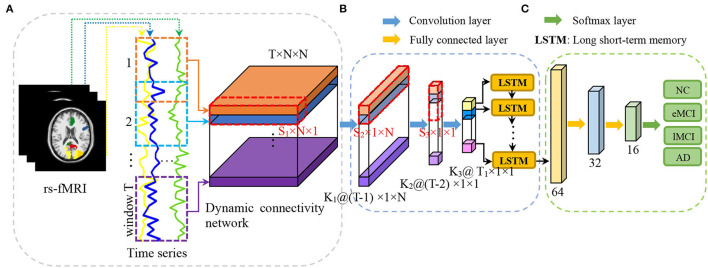
Illustration of the proposed convolutional recurrent neural network (CRNN) for brain disease classification with rs-fMRI data, consisting of **(A)** image preprocessing and dynamic functional connectivity network construction, **(B)** temporal and sequential features extraction *via* three convolutional layers and a long short-term memory (LSTM) layer, and **(C)** classification *via* three fully connected layers.

In this article, we propose a convolutional recurrent neural network (CRNN) for brain disease classification with rs-fMRI data, which can explicitly model the sequential information conveyed in dynamic FC networks for end-to-end brain disease identification. To the best of our knowledge, this is among the first attempt to construct a convolutional recurrent neural network for dFC network analysis using rs-fMRI data. Figure 1 illustrates the architecture of our proposed CRNN method. Specifically, we first construct dFC networks from the rs-fMRI time series at successive, overlapping time windows, where Pearson correlation coefficients (PCC) of the region-based BOLD signals are used as measures of FC between brain regions. Then, we employ three convolutional layers to extract features from the constructed dFC networks and construct a long short-term memory (LSTM) (Sainath et al., 2015) layer to capture sequential information of extracted features. The extracted features are finally fed into three fully connected layers to identify patients with brain diseases from normal controls. We evaluated the proposed CRNN method on 174 subjects with 563 rs-fMRI scans from the Alzheimer's Disease Neuroimaging Initiative (ADNI) database^1^. The experimental results demonstrate the effectiveness of our proposed method.

The remainder of this article is organized as follows. In section 2, we first review related study on the FC network and deep learning for brain disease diagnosis. We then introduce the materials used in the experiments and the details of our proposed framework in section 3. In section 4, we first introduce the experimental setup and methods for comparison and then present the corresponding experimental results. In section 5, we study the influence of key parameters and point out several limitations of this study as well as future research directions. Finally, we conclude this article in section 6.

## 2. Related Study

### 2.1. Brain Network Analysis

Network analysis provides an important way of exploring the association between brain functional organization and brain diseases (Kaiser, 2011). Learning properties of brain networks shows great promise for identifying biomarkers from neuroimaging data. Functional connectivity (FC) networks estimated from rs-fMRI have been extensively used for brain disease analysis. Many FC network analysis models have been developed, from simple stationary FC (sFC) networks (Zanin et al., 2012; Qiao et al., 2018) to complex time-frequency analysis based dynamic FC (dFC) networks (Jones et al., 2012; Jie et al., 2018). Previous studies have found disrupted FC associated with specific brain regions in patients with AD/MCI such as the hippocampus (Liu et al., 2008), posterior cingulate cortex (Bai et al., 2009), and amygdala (Yao et al., 2013), and have reported abnormal network properties such as changed small world characteristics in AD/MCI patients (Supekar et al., 2008). However, these studies mainly focus on topological differences of FC networks between patients and normal controls (NCs) using graph theory (Stam et al., 2009; Brier et al., 2014).

On the other hand, several studies have successfully applied FC networks to the classification of brain diseases using machine learning methods. For example, Jie et al. (2014) used a graph-kernel-based method to identify patients with MCI from normal controls. Wee et al. (2012) designed a multimodality classification framework based on a multi-kernel support vector machine (SVM). Gan et al. (2021) developed a multi-graph fusion method to fuse FC networks, and employed sparse-SVM to jointly conduct brain region selection and disease diagnosis. Several studies have integrated multiple properties of FC networks for the diagnosis of MCI (Zanin et al., 2012; Jie et al., 2018), autism (Anderson et al., 2011), and early tourette syndrome in children (Wen et al., 2017). In general, existing studies on FC network classification mainly rely on two kinds of methods: traditional machine learning methods and deep learning methods, with details introduced as follows.

### 2.2. Machine Learning for Brain Network Classification

In traditional machine learning based methods, many handcrafted measures are usually extracted from FC networks as features for training a classification model (e.g., support vector machine, SVM). For example, several studies extracted connectivity strength (Chen et al., 2011) and local clustering coefficient (Wee et al., 2012; Jie et al., 2016) from sFC network as features to train an SVM for MCI identification. Additionally, some studies extracted and integrated multiple properties of sFC networks for the diagnosis of brain diseases (Zanin et al., 2012; Jie et al., 2013), and achieved better performance in comparison with single network measures.

On the other hand, temporal dynamics of dFC networks are extracted for brain disease analysis (Chang and Glover, 2010). For example, several studies have extracted clustering coefficients (Wee et al., 2016), temporal variabilities (Sakoğlu et al., 2010; Jie et al., 2018), and/or root-mean-square (Chen et al., 2017) features from dFC networks to train a classifier for brain disease classification, and achieve better classification performance compared with sFC network based methods. However, these studies generally treat feature learning and classification separately, which could degrade the classification performance. Also, these studies typically extract handcrafted network measures as features, which highly rely on expert knowledge.

### 2.3. Deep Learning for Brain Network Classification

Deep learning methods (e.g., convolutional neural networks, CNNs) that can learn representations of data have been successfully applied to various fields (LeCun et al., 2015). There is significant interest in the development and application of deep learning methods to medical image analysis, including image segmentation (Milletari et al., 2016; Akkus et al., 2017), registration (Boveiri et al., 2020), and brain disease diagnosis (Shen et al., 2015; Deepak and Ameer, 2019; Wen et al., 2020). In recent years, many studies have successfully applied deep learning methods to brain FC network analysis (Ju et al., 2017; Zeng et al., 2018; Zhang et al., 2020). For example, Kawahara et al. (2016) developed a CNN-based FC network analysis method to predict cognitive and motor developmental outcome scores of preterm infants. Zeng et al. (2018) built a deep discriminant autoencoder network for cross-site classification of schizophrenia. Meszlényi et al. (2017) developed a CNN-based method for FC network analysis and MCI classification. He et al. (2020) developed a deep neural network (DNN) for FC prediction of behavior and demographics Unfortunately, these methods mainly focus on stationary FC networks and, thus, cannot be applied to dynamic FC network analysis.

Several recent studies employ deep learning methods for dFC network based disease analysis and yield better performance compared with sFC network based methods. For example, Wang et al. (2019a) presented a dFC network-based CNN framework for electroencephalogram (EEG) based person identification in diverse human states. Jie et al. (2020) built a weighted correlation kernel based CNN framework for automated diagnosis of MCI. However, the valuable sequential information conveyed in dynamic FC networks is generally neglected in these studies. To this end, we propose a convolutional recurrent neural network to explicitly capture the sequential information conveyed in dynamic FC networks for brain disease classification with rs-fMRI data.

## 3. Materials and Methods

Figure 1 illustrates the proposed convolutional recurrent neural network (CRNN) for rs-fMRI based brain disease classification, consisting of three parts: (a) image preprocessing and dynamic functional connectivity (dFC) network construction, (b) feature learning, and (c) classification. In this section, we first present the subjects used in this study and then introduce the details of the proposed method.

### 3.1. Subjects

The rs-fMRI data obtained from the ADNI database were studied in this paper. In this study, we used 563 scans of 174 subjects, including 31 AD, 45 late MCI (lMCI), 50 early MCI (eMCI), and 48 normal controls (NCs). It is worth noting that the subjects participating in the study may have one or more scans at different time points. The 563 scans can be divided into 99 cases of AD, 145 cases of lMCI, 165 cases of eMCI, and 154 cases of NC. The specifications of the data acquired in each scan are: the in-plane image resolution is 2.29−3.31*mm*, the slice thickness is 3.31*mm*, TE (echo time) is 30*ms*, and TR (repetition time) is 2.2−3.1*s*. There are 140 volumes (time points) for each subject. The demographic and clinical information of rs-fMRI scans of all subjects is summarized in Table 1.

**Table 1:** Demographic information of all rs-fMRI scans of the subjects used in this study (Mean ± SD).

**Group**	**AD**	**lMCI**	**eMCI**	**NC**
Gender (M/F)	55/44	95/50	73/92	67/87
Age (Years)	74.7 ± 7.4	72.3 ± 8.1	72.4 ± 7.1	76.0 ± 6.8
MMSE	21.8 ± 3.3	27.1 ± 2.1	28.1 ± 1.6	28.8 ± 1.4

*M/F, Male/Female*.

*MMSE, Mini-Mental State Examination*.

### 3.2. Proposed Method

#### 3.2.1. Image Preprocessing and Network Construction

All rs-fMRI data are preprocessed by standard procedures using FSL FEAT software. Specifically, we discard the first 3 volumes before preprocessing, and then process the remaining 137 volumes according to the standard pipeline, including (1) slice timing correction, (2) head motion estimation, (3) bandpass filtering, and (4) the regression of white matter, cerebrospinal fluid and motion parameters. To reduce the influence of head motion, we remove the fMRI data of the head moving more than 2.0*mm* in any direction or 2° in any rotation. Then, we perform structural skull stripping and map the fMRI data of the skull stripping to the Montreal Neurological Institute space. A 6*mm* Gaussian kernel is used to spatially smooth the rs-fMRI data. Subjects whose frame displacement exceeds 2.5 min (FD >0.5) are not included in our analysis. Finally, using the automated anatomical labeling (AAL) template, the brain space of each subject's fMRI scan was partitioned into 116 regions of interest (ROIs). For each subject, the average rs-fMRI time series from the BOLD signals in all ROIs were calculated, which are used for the construction of dFC networks.

We constructed the dFC network using an overlapping sliding window strategy. As shown in Figure 1A, for each subject with *N* ROIs, we first partition the time series into *T* overlapped windows/segments, and build an FC network/matrix G^*t*^∈ℝ^*N*×*N*^ within the *t*-th (*t* = 1, ⋯ , *T*) time window. In G^*t*^, each node denotes a specific ROI, and the edge weight between a pair of ROIs is the Pearson's correlation coefficient (PCC). In this way, we can obtain a set of *T* FC networks 𝒢 with the window length of *L* and the sliding step size of *S* to characterize the dynamics of brain networks for each subject, i.e., 𝒢 = [G^1^, G^2^, ⋯ , G^*T*^]∈ℝ^*T*×*N*×*N*^.

#### 3.2.2. Temporal and Sequential Feature Extraction

As shown in Figure 1B, we define four layers in the proposed CRNN framework to model both temporal and sequential features of the constructed dynamic functional connectivity networks. Specifically, following (Jie et al., 2020), we first define three convolutional layers to extract hierarchical (i.e., region, whole-network, and temporal) features from the constructed dFC networks, respectively. Then, we use a recurrent neural network layer to learn the important sequential features of brain networks. More details can be found in the following.

*(i) Region feature extraction*. To learn region features from the whole dFC networks, in the first convolutional layer, we set the size of the kernel of *S*_1_ × *N* × 1 and set the stride along three dimensions (i.e., one temporal and two spatial dimensions) to (1, 1, 1). The convolution along two spatial dimensions is a feature mapping for each ROI, while the convolution along the temporal dimension denotes different feature mappings of the same ROI. This helps characterize the temporal properties of the corresponding ROI. Features extracted from this layer characterize temporal dynamics of brain regions, and these features are high-order since these features are calculated based on functional connectivities of specific ROI across *S*1 FC networks.

*(ii) Whole-network feature extraction*. To extract whole-network features from our learned region features, in the second convolutional layer, we set the kernel of *S*_2_ × 1 × *N*, and set the stride in three dimensions to (1, 1, 1). For this layer, the convolution along two spatial dimensions is a feature mapping for the whole FC network. The convolution along the temporal dimension represents different mappings of the whole FC network, reflecting temporal changes in dFC networks.

*(iii) Temporal feature extraction*. We further define the third convolutional layer to model the temporal features of the whole dFC network. Specifically, we set the kernel size of *S*_3_ × 1 × 1 and set the stride size in three dimensions to (2, 1, 1). Therefore, the features obtained in this layer with a kernel can be considered representations of the temporal dynamics of the brain network. It is worth noting that these three convolution layers are used for learning high-level and high-order temporal features from dFC networks derived from the rs-fMRI time series.

*(iv) Sequential feature extraction*. As a type of RNN that incorporates a memory cell, long short-term memory (LSTM) (Sainath et al., 2015) has been successfully applied to temporal modeling in various domains, such as video and speech analysis. To capture the temporal dynamics of brain networks, we propose to use an LSTM layer to model the sequential information of dFC networks. Specifically, to fit the input shape required by the LSTM, we first flatten the temporal features learned in the previous convolutional layer. The flattened features are then fed into an LSTM layer defined as follows:


(1) $$\begin{array}{ll} f^{t}=\sigma \left(U^{f}x^{t}+W^{f}h^{t-1}+b^{f}\right)\; & \; \end{array}$$

(2) $$\begin{array}{l} g^{t}=\tanh\left(U^{g}x^{t}+W^{g}h^{t-1}+b^{g}\right) \end{array}$$

(3) $$\begin{array}{l} o^{t}=\sigma \left(U^{o}x^{t}+W^{o}h^{t-1}+b^{o}\right) \end{array}$$

(4) $$\begin{array}{l} s^{t}=f^{t}s^{t-1}+g^{t}\sigma \left(U^{i}x^{t}+W^{i}h^{t-1}+b^{i}\right) \end{array}$$

(5) $$\begin{array}{l} h^{t}=\tanh\left(s^{t}\right)o^{t} \end{array}$$

where *f*^*t*^, *g*^*t*^, and *o*^*t*^ denote the forget gate unit, the external input gate unit, and the output gate unit at time *t*, respectively. Here, *x*^*t*^, *h*^*t*^, and *s*^*t*^ are the input vector, hidden vector, and state vector, respectively. *U*^*k*^, *W*^*k*^, and *b*^*k*^ with *k*∈*f, g, o, i* denote weights and biases, respectively. Additionally, σ and tanh denote sigmoid and hyperbolic tangent activation functions. The output sequential features of the LSTM layer are subsequently reshaped back to feed the next layer for classification.

#### 3.2.3. Classification

In the classification module, we use three fully connected layers (containing 32, 16, and 4 neurons, respectively) to learn a mapping between sequential features and category labels (e.g., patient with AD or NC) for classification. The output (*via* softmax) of the proposed method is the probability of the subject belonging to a specific category. In the proposed CRNN, a rectified linear unit (ReLU) is employed as the activation function of each layer, and dropout with a rate of 0.25 is used in each fully connected layer.

#### 3.2.4. Implementation

The proposed CRNN is implemented in Python based on the Keras package, and the model is trained on a single GPU (NVIDIA GeForce GTX 1080Ti) with 11 GB of memory. When constructing dynamic FC networks, we empirically set the fixed length of the time window as *L* = 70 and the sliding step size as *S* = 2. Therefore, the number of time windows per subject is *T* = 34. In the three convolutional layers, we, respectively, set the convolution kernel size along the time dimension as follows: *S*_1_ = 2, *S*_2_ = 2, and *S*_3_ = 8. The corresponding channel numbers of the three convolutional layers are set as follows: *K*_1_ = 8, *K*_2_ = 16, and *K*_3_ = 32. According to the above parameters, we can calculate *T*_1_ = 13. When predicting the progression of brain diseases, softmax is used as the activation function of the last fully connected layer for binary classifications and four-category classifications. Here, we use the Adam optimizer with recommended parameters for training, by empirically setting the number of epochs to 200 and the batch size to 16.

## 4. Experiment

### 4.1. Experimental Settings

We employ a *subject-level* 5*-fold cross-validation* strategy in this study, ensuring that scans from the same subject do not appear in both train and test sets. Specifically, all subjects with baseline were first divided into 5 subsets of roughly the same size. Each subset is then selected in turn as the test set, and the remaining 4 subsets (including their other scans) and subjects without baseline scans are combined to form the training set. In addition, in each fold of cross-validation, we further select 20% of the training data as the validation data to determine the optimal parameters of a specific classification model. It is worth noting that we only use a baseline scan of each subject in the test set as testing subject to evaluate the performance of our proposed method. Also, each scan of each subject is considered an independent sample, but all scans of the same subject have the same category label. Finally, the classification results generated by a method with 5-fold cross-validation are averaged and recorded.

In order to evaluate the effectiveness of the proposed model, we conducted both binary and multi-class classification experiments, including (1) eMCI vs. NC classification, (2) AD vs. NC classification, and (3) AD vs. lMCI vs. eMCI vs. NC classification. Three evaluation metrics are used to measure the performance of binary classifications, namely classification accuracy (ACC), sensitivity (SEN), and specificity (SPE). For multi-class disease classification, we evaluate the performance by calculating both the overall accuracy of all categories and the classification accuracy of each category.

### 4.2. Methods for Comparison

In the experiments, we first compared our method with three baseline methods and state-of-the-art methods.

(1) **baseline**: In this method, we first construct an sFC network for each subject by calculating the Pearson correlation coefficient between the entire time series of any pair of ROIs. Then, the connectivity strength of the stationary FC network is used as a network feature. The *t*-test method with a threshold (i.e., *p*-value < 0.05) is used for feature selection of binary class tasks. Finally, a linear support vector machine (SVM) with default parameters is used for classification.

(2) **CC**: In this method, the subject's stationary FC network is first constructed. Then the local clustering coefficients of all 116 ROIs from the constructed stationary FC network are extracted as features. We use a *t*-test and linear SVM with default parameters for feature selection and classification, respectively.

(3) **M**^**2**^**TFS**: Similar to our proposed CRNN, this method first constructs a set of *T* dynamic FC networks for each subject. Then, following (Jie et al., 2018), the temporal and spatial mean features of the dynamic FC network are extracted. The manifold regularized multi-task feature selection (M^2^TFS) method and multi-kernel SVM are used for feature selection and classification, respectively.

(4) **CNN**: This method is a state-of-the-art approach for rs-fMRI analysis. For a fair comparison, the CNN method employs the same architecture as our method, but without using recurrent neural networks to extract sequential features. That is, CNN does not use the LSTM layer, but instead uses an average pooling layer, followed by three fully connected layers for classification.

### 4.3. Classification Performance

**Figure 2:**
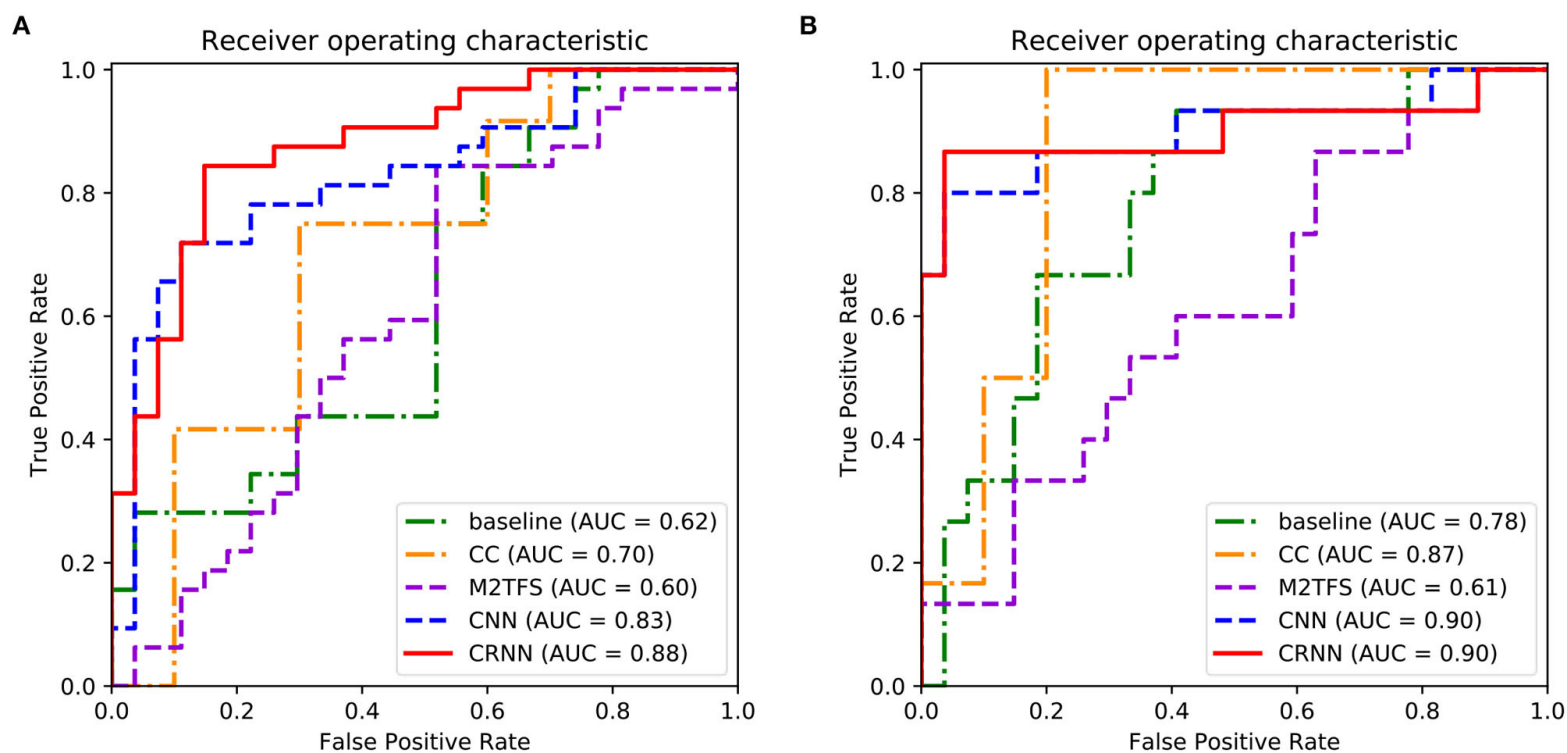
Receiver operating characteristic (ROC) curves achieved by five different methods in **(A)** eMCI vs. NC classification and **(B)** AD vs. NC classification.

Tables 2, 3 report the quantitative results of different methods in two binary classifications and a multi-class classification task, respectively. The receiver operating characteristic (ROC) curves of two binary classification tasks are plotted in Figure 2. It can be seen from Figure 2 and Tables 2, 3 that our proposed CRNN method is generally better than the four competing methods in the three classification tasks. For example, the ACC values of our proposed CRNN in eMCI vs. NC classification and AD vs. NC classification are 84.5 and 92.8%, respectively, while the second best ACC results obtained by CNN are 76.2 and 87.8%, respectively. For the challenging AD vs. lMCI vs. eMCI vs. NC classification task, the overall accuracy of our CRNN is 61.7%, which is an increase of 8.9% compared with CNN. These results indicate that our proposed CRNN method is effective in predicting the progression of brain diseases based on rs-fMRI.

**Table 2:** Performance of five methods in two binary classification tasks, i.e., eMCI vs. NC and AD vs. NC classifications (Mean ± SD).

**Method**	**eMCI vs. NC (%)**				**AD vs. NC (%)**		
	**ACC**	**SPE**	**SEN**	**ACC**	**SPE**	**SEN**
baseline	57.1 ± 0.4	48.1 ± 11.5	65.6 ± 11.3	73.3 ± 12.6	77.8 ± 1.9	66.7 ± 0.0
CC	63.6 ± 5.7	50.0 ± 14.1	75.0 ± 15.4	75.0 ± 15.4	80.0 ± 8.3	66.7 ± 17.1
M^2^TFS	67.7 ± 2.0	47.3 ± 5.8	84.7 ± 1.4	76.4 ± 5.8	**100.0 ± 0.0**	33.3 ± 19.2
CNN	76.2 ± 8.1	77.3 ± 7.7	75.2 ± 19.2	87.8 ± 6.9	92.0 ± 11.5	80.0 ± 19.2
CRNN (ours)	**84.5 ± 4.7**	**84.0 ± 11.5**	**84.8 ± 14.3**	**92.8 ± 6.9**	96.7 ± 0.0	**86.7 ± 18.3**

*The bold values indicate the one with the largest mean among all methods*.

**Table 3:** Performance of five methods in the multi-class classification task, i.e., AD vs. lMCI vs. eMCI vs. NC classification.

**Method**	**AD vs. lMCI vs. eMCI vs. NC (%)**				
	**ACC**	**ACC_*NC*_**	**ACC_*eMCI*_**	**ACC_*lMCI*_**	**ACC_*AD*_**
Baseline	30.6 ± 9.2	20.0 ± 7.8	38.9 ± 18.5	30.0 ± 6.8	33.3 ± 13.6
CC	35.0 ± 10.5	22.0 ± 11.9	69.5 ± 16.2	21.0 ± 17.4	6.7 ± 14.9
M^2^TFS	44.0 ± 1.2	36.0 ± 20.4	**87.6 ± 9.6**	22.0 ± 28.9	0.0 ± 0.0
CNN	52.8 ± 10.8	44.7 ± 30.6	53.8 ± 4.1	65.0 ± 8.7	46.7 ± 19.2
CRNN (ours)	**61.7 ± 2.8**	**65.3 ± 3.8**	63.3 ± 5.5	**65.0 ± 12.6**	**46.7 ± 19.2**

*ACC, Accuracy. The bold values indicate the one with the largest mean among all methods*.

In addition, from Figure 2 and Tables 2, 3, we can also have three interesting observations. *First*, methods based on dynamic FC networks (i.e., M^2^TFS, CNN, and CRNN) are generally superior to methods based on static FC networks (i.e., baseline and CC). This suggests that the dynamic changes of the rs-fMRI time series can provide useful information for a better understanding of the pathology of brain diseases. *Second*, compared with traditional machine learning methods (i.e., baseline, CC, and M^2^TFS), deep learning methods (i.e., CNN and CRNN) can achieve better performance. This shows that deep learning can capture the potential discriminative features of brain networks. *Finally*, compared with the CNN method, our CRNN can obtain better performance, which proves the advantage of mining sequential features from a dynamic FC network.

**Figure 3:**
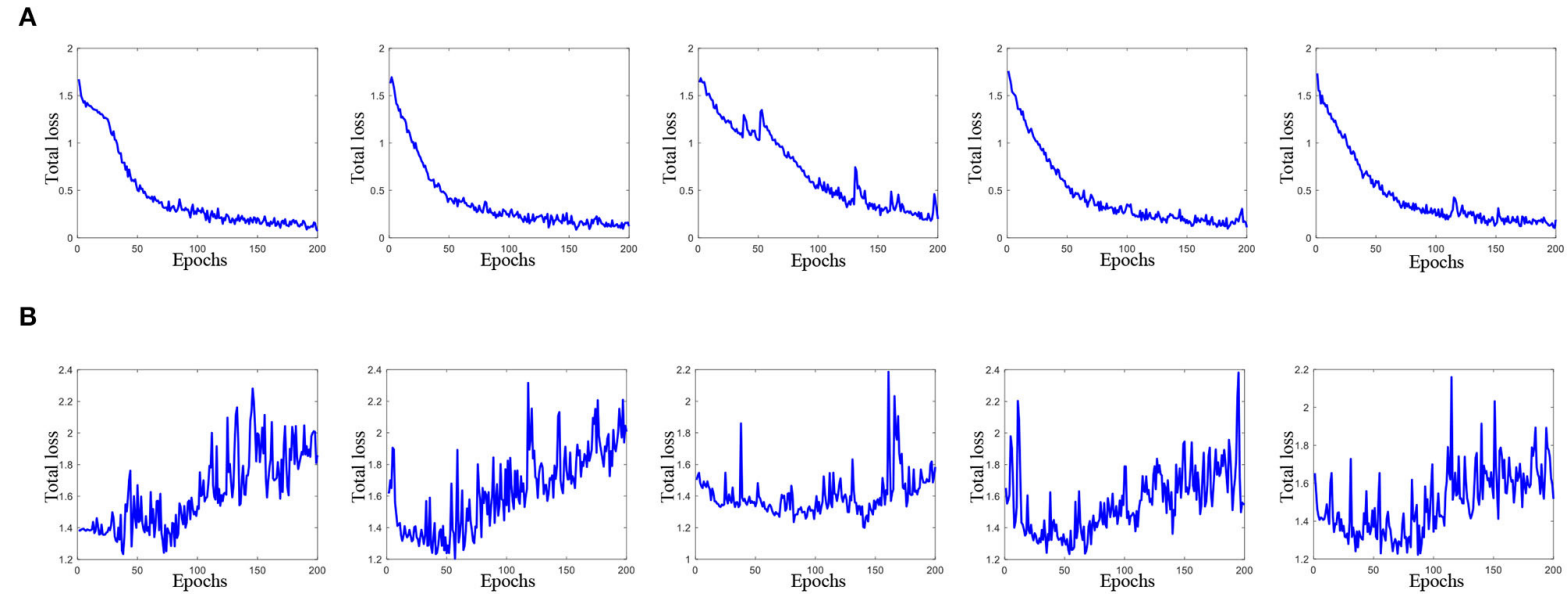
Total loss of the proposed method with 200 epochs in each fold cross-validation (from left to right) for AD vs. lMCI vs. eMCI vs. NC classification task. Here, **(A)** total loss of training data, and **(B)** total loss on validation data.

On the other hand, Figure 3 plots the total loss curve of training subjects and validation subjects in each fold of cross-validation for the task of AD vs. lMCI vs. eMCI vs. NC classification. It can be seen from Figure 3 that our proposed CRNN method can quickly converge within 80 epochs.

### 4.4. Discriminative Functional Connectivity

We further conduct experiments to identify discriminative brain regions that contribute the most to a specific classification task and identify the informative functional connectivity between discriminative brain regions.

Specifically, in our proposed CRNN method, the output of the first convolutional layer is a 4-dimensional tensor of size (*T*−1) × 1 × *N* × *K*_1_, representing the feature vector of each subject in *T*−1 time series segments. For simplicity, we average the feature vectors of *T*−1 time series segments. Since there are *K*_1_ = 8 channels in the first convolutional layer, we can construct 8 feature vectors for each subject, and each feature vector corresponds to a specific channel. Then, the standard *t*-test was used to measure the group difference between eMCI vs. NC and AD vs. NC, respectively. It is worth noting that the feature vector obtained in this way may be different in each fold of cross-validation. For each channel, we use the standard *t*-test for each fold cross-validation to integrate all brain regions with *p*-values less than 0.05 in 5-fold cross-validation, and select brain regions that appear 3 times or more as discriminative brain regions.

**Figure 4:**
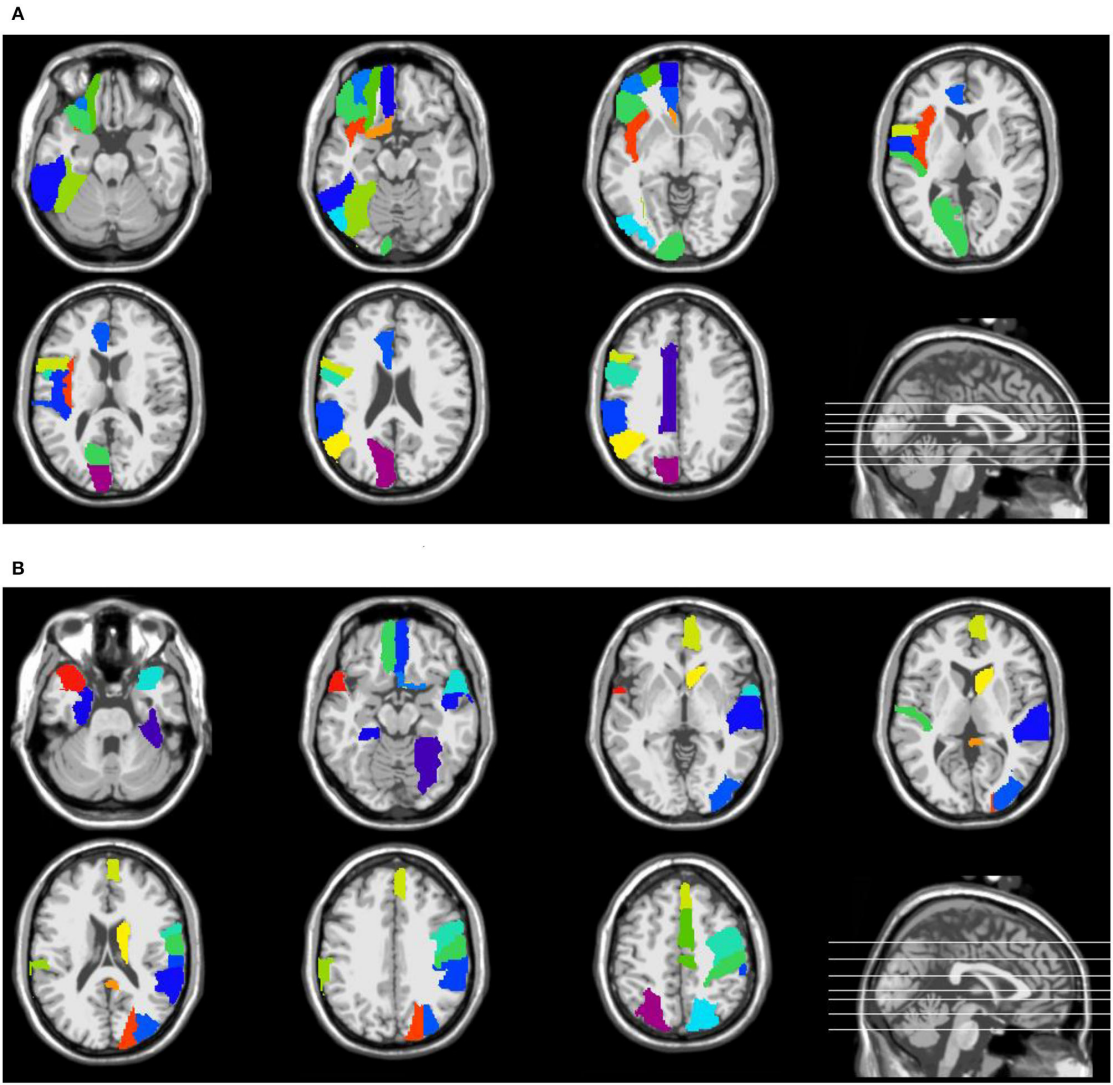
Discriminative brain regions identified by the proposed method in **(A)** eMCI vs. NC classification and **(B)** AD vs. NC classification.

Supplementary Tables S1, S2 in the Supporting Information list the names and abbreviations of discriminative brain regions in the eMCI vs. NC group and AD vs. NC group, respectively. Figure 4 plots those discriminative brain regions in the template space. From Figure 4 and Supplementary Tables S1, S2, we first can see that) the discriminative brain regions of the two groups (i.e., eMCI vs. NC group and AD vs. NC group) all contain the cerebellum, indicating that the cerebellum may be related to MCI/AD and may provide useful information for brain diseases prognosis (Thomann et al., 2008). Second, the discriminative brain regions in the eMCI vs. NC group are largely located in the left hemisphere. We speculate that during the evolution of AD, the left half part of some brain regions of eMCI patients may first begin to have brain atrophy, which leads to changes in their functional connectivity (Thompson et al., 2003; Daianu et al., 2013). Finally, the discriminative brain regions selected by our proposed method in two classification tasks are consistent with previous studies. For example, the brain regions detected in the AD vs. NC classification, including posterior cingulate gyrus, parahippocampal gyrus, fusiform gyrus, and temporal pole, have been reported to be useful in discriminating patients with AD from NCs (Jie et al., 2020). Also, the precentral gyrus identified by our method processes auditory error signals during speech production to maintain fluency (Ozker et al., 2022), and the orbitofrontal cortex plays an important role in decision-making and emotion processing (Bechara et al., 2000; Rolls, 2004). Abnormal changes in these regions can lead to the development of brain disease and also suggest that the discriminative brain regions identified by our method are associated with MCI/AD.

**Figure 5:**
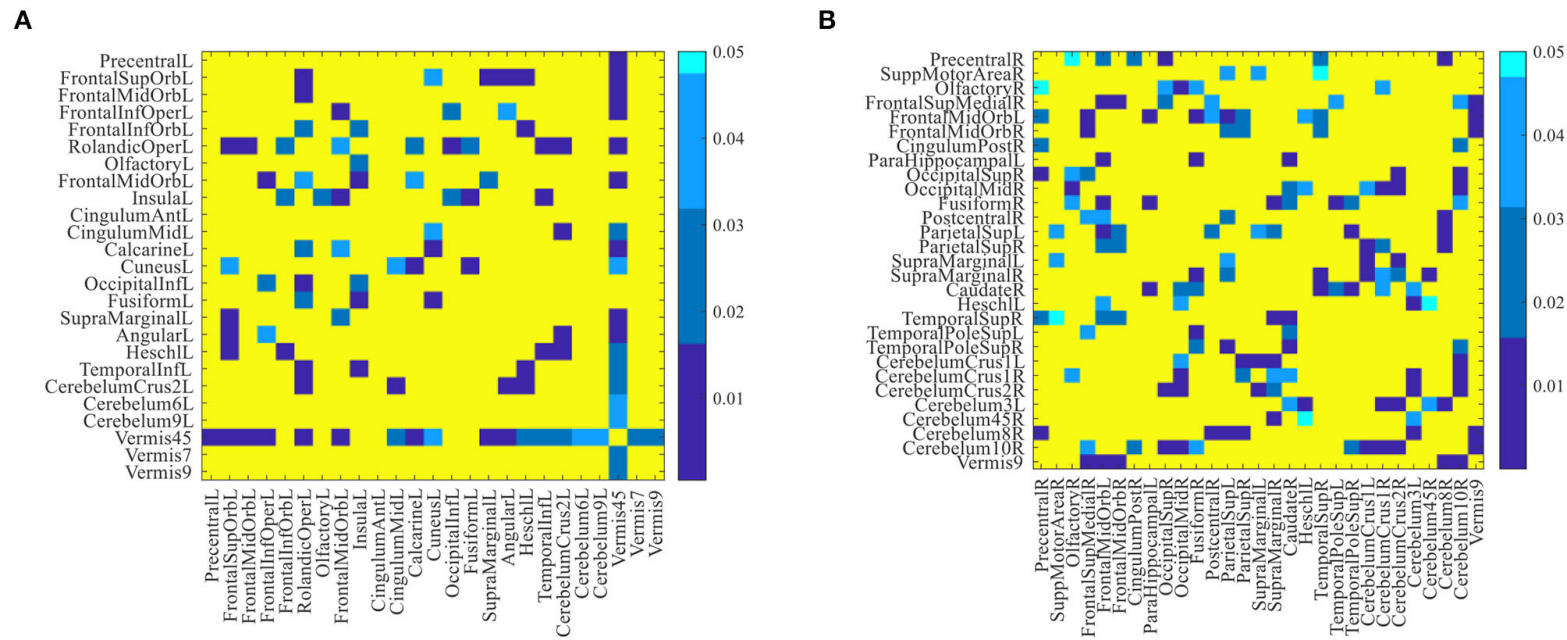
The difference in connectivity strength between the discriminative brain regions for **(A)** eMCI vs. NC group and **(B)** AD vs. NC group. Here, *p*-values larger than 0.05 are set to 1 (corresponding to the yellow part in the figure).

Furthermore, we performed a standard *t*-test on the functional connectivity between selected discriminative brain regions. Figure 5 plots the group difference in the connectivity strength between the discriminative brain regions in the eMCI vs. NC group and the AD vs. NC group in Supplementary Tables S1, S2. Here, the color indicates the corresponding *p*-value, we set *p*-values more than 0.05 to 1 for clarity. From Figure 5, we can clearly observe that there are strong correlations between the two brain regions of lobules IV and V of the vermis and the left rolandic operculum with other discriminative brain regions in the eMCI vs. NC group. In addition, we can also observe that compared with the eMCI vs. NC group, the AD vs. NC group has more connectivity strength with *p*-values less than 0.05 (corresponding to the blue part in Figure 5). This suggests that the change in the connectivity strength in the AD vs. NC group is more pronounced than that in the eMCI vs. NC group, reflecting that AD's impairment of the brain gradually increases as the disease progresses.

**Figure 6:**
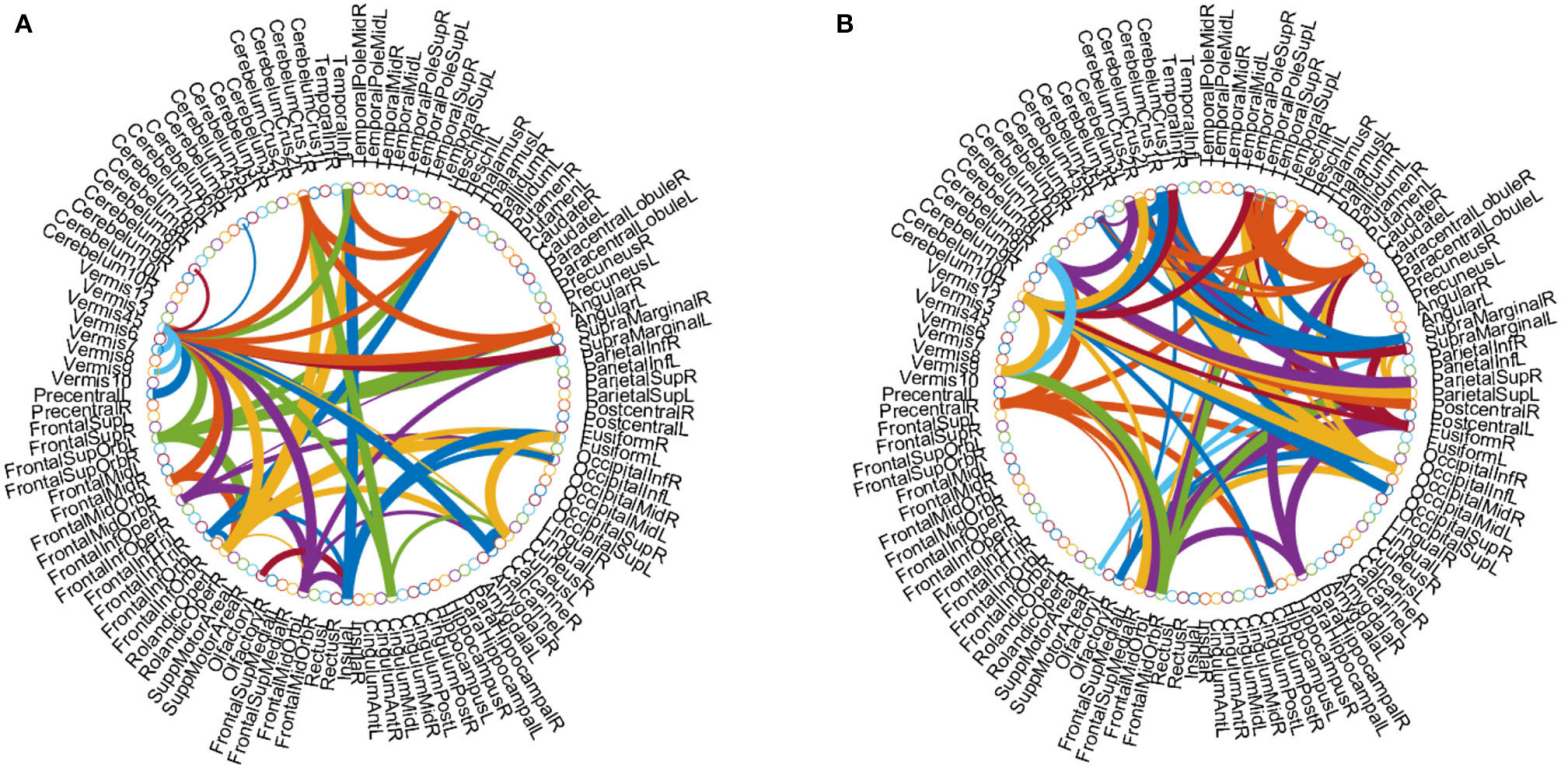
Discriminative functional connectivities for **(A)** eMCI vs. NC and **(B)** AD vs. NC classification. Each arc shows the selected connectivity between two ROIs, where colors are randomly allocated for better visualization and the thickness of each arc indicates its discriminative power that is inversely proportional to the corresponding *p*-value in the *t*-test.

Besides, we identified the functional connectivities with *p*-values less than 0.05 between discriminative brain regions as the most discriminative functional connectivity. Figure 6 plots the most discriminative functional connectivities on the 5th and 7th channels for eMCI vs. NC group and AD vs. NC group. As shown in Figure 6, for eMCI vs. NC classification, the brain regions we selected include the left fusiform gyrus, the left lobule VI of the cerebellar hemisphere, and lobule VII of the vermis. The functional connectivity of these brain regions is significantly reduced in MCI patients, which is consistent with previous studies by Bokde et al. (2006) and Thomann et al. (2008). For AD vs. NC classification, there are two discriminative brain regions selected by our method, including the left crus I of the cerebellar hemisphere and the right lobule IV, V of the cerebellar hemisphere. According to previous studies by Thomann et al. (2008), these two brain regions may be biologically related to AD. These results further confirm that our method is potentially helpful in discovering fMRI biomarkers for MCI and AD identification.

### 4.5. Discriminative Power of Learned Features

**Figure 7:**
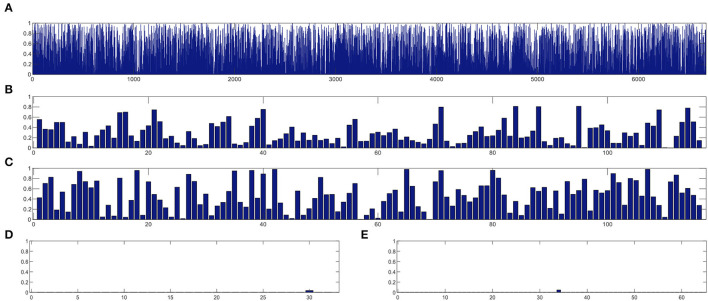
The discriminative power of features between eMCI and NC groups learned by **(A)** baseline, **(B)** CC, **(C)** M^2^TFS, **(D)** CNN, and **(E)** CRNN.

**Figure 8:**
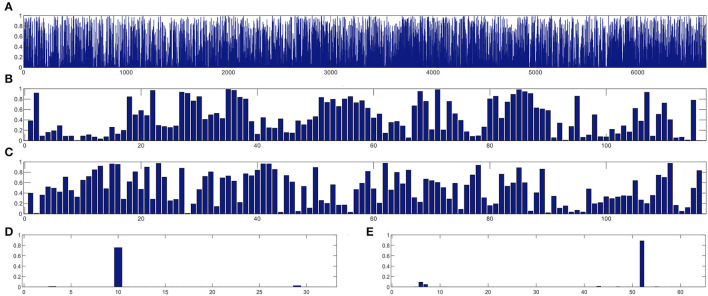
The discriminative power of features between AD and NC groups learned by **(A)** baseline, **(B)** CC, **(C)** M^2^TFS, **(D)** CNN, and **(E)** CRNN.

In this section, we study the discriminative power of the features learned in our proposed CRNN method. Specifically, in the multi-class classification task, we first extract the learned sequential features from the model (corresponding to the output of the LSTM layer in our proposed CRNN method). Here, each sequential feature learned is a feature mapping of the dynamic FC network with respect to time changes. Then, we use the standard *t*-test to calculate the discriminative power of all sequential features in the eMCI vs. NC group and AD vs. NC group, with *p*-values shown in Figures 7, 8, respectively. For comparison, we also report the discriminative power of the features learned by the comparison methods (i.e., baseline, CC, M^2^TFS, and CNN). There are a total of 6,670 connectivity strength features in the baseline method, 16 features in SVM-based methods (i.e., CC and M^2^TFS), 32 features in CNN, and 64 features in our CRNN, respectively. In addition, Table 4 reports the average and median values of *p*-values for all features in both group pairs.

**Table 4:** The average and median of *p*-value for learning features in both group pairs (i.e., eMCI vs. NC groups, and AD vs. NC groups).

**Method**	**eMCI vs. NC**			**AD vs. NC**	
	**Mean**	**Median**		**Mean**	**Median**
Baseline	0.38	0.32	0.36	0.28	
CC	0.29	0.24	0.44	0.40	
M^2^TFS	0.47	0.48	0.49	0.48	
CNN	0.01	0	0.02	0	
CRNN	<1E−3	0	0.01	<1E−33	

From Figures 7, 8 and Table 4, we can observe that the *p*-values of the features learned by the CNN and CRNN methods are mostly close to 0 (i.e., very sparsity) compared to other methods. It implies that both methods are capable of distinguishing patients from NCs. Second, the *p*-value of our proposed CRNN method is more sparsity than that of the CNN method, indicating the features learned by the CRNN method are more discriminative than those learned by other methods, which also explains why our method can achieve better classification performance. The above results further demonstrate the effectiveness of the sequential features extracted by our CRNN method.

## 5. Discussion

### 5.1. Effect of Sliding Window Parameters

**Figure 9:**
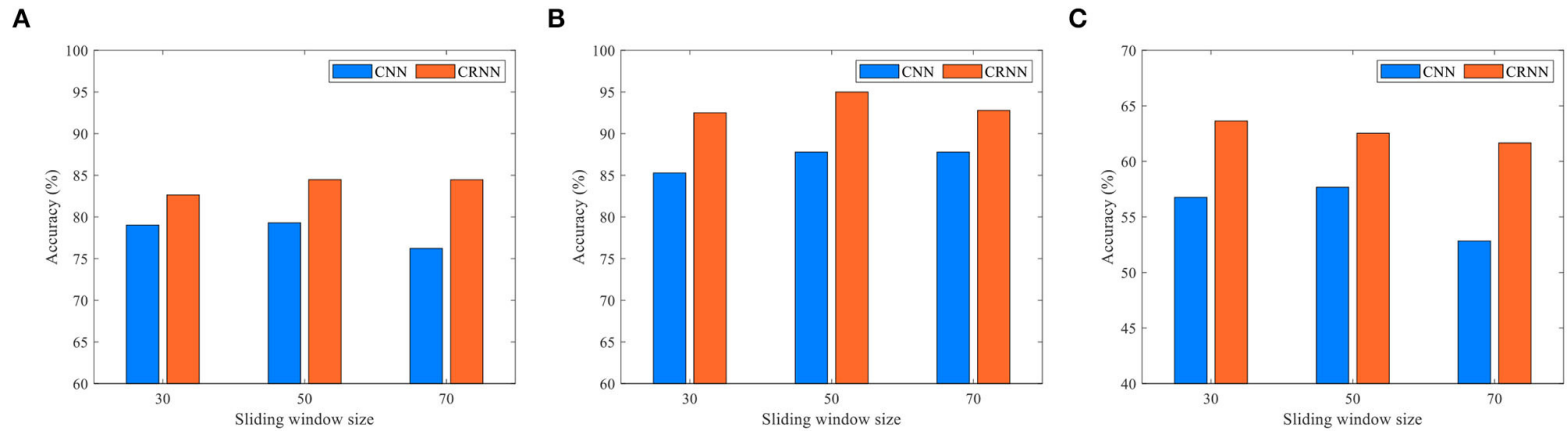
Classification accuracy of the CRNN and CNN methods using different lengths of sliding windows in three tasks: **(A)** eMCI vs. NC classification, **(B)** AD vs. NC classification, and **(C)** AD vs. lMCI vs. eMCI vs. NC classification.

Existing studies have shown that FC time series based on rs-fMRI have regular temporal variability (Huang et al., 2016). In our proposed CRNN, we use the overlapping sliding window technology (stride: 2) to divide the time series, where the parameter *L* determines the length of the time window in the construction of a dynamic FC network. To investigate the effects of the sliding time window length on the classification performance of our proposed CRNN, we set the parameters *L* to 30, 50, and 70 for classification, respectively. In Figure 9, we report the classification accuracy of the CRNN method under different *L* values on two binary classification tasks and one four-class classification task (i.e., eMCI vs. NC group, AD vs. NC group, and AD vs. lMCI vs. eMCI vs. NC group), respectively. At the same time, we also report the classification accuracy of CNN using different sliding window lengths.

As shown in Figure 9, for all given parameter values, our proposed CRNN method outperforms CNN on the three classification tasks, which further illustrates the effectiveness of the sequential features extracted by our method. In addition, the classification accuracy of the proposed CRNN on the three classification tasks is not greatly affected by the different values of *L*, which shows that our method has strong robustness and stability.

**Figure 10:**
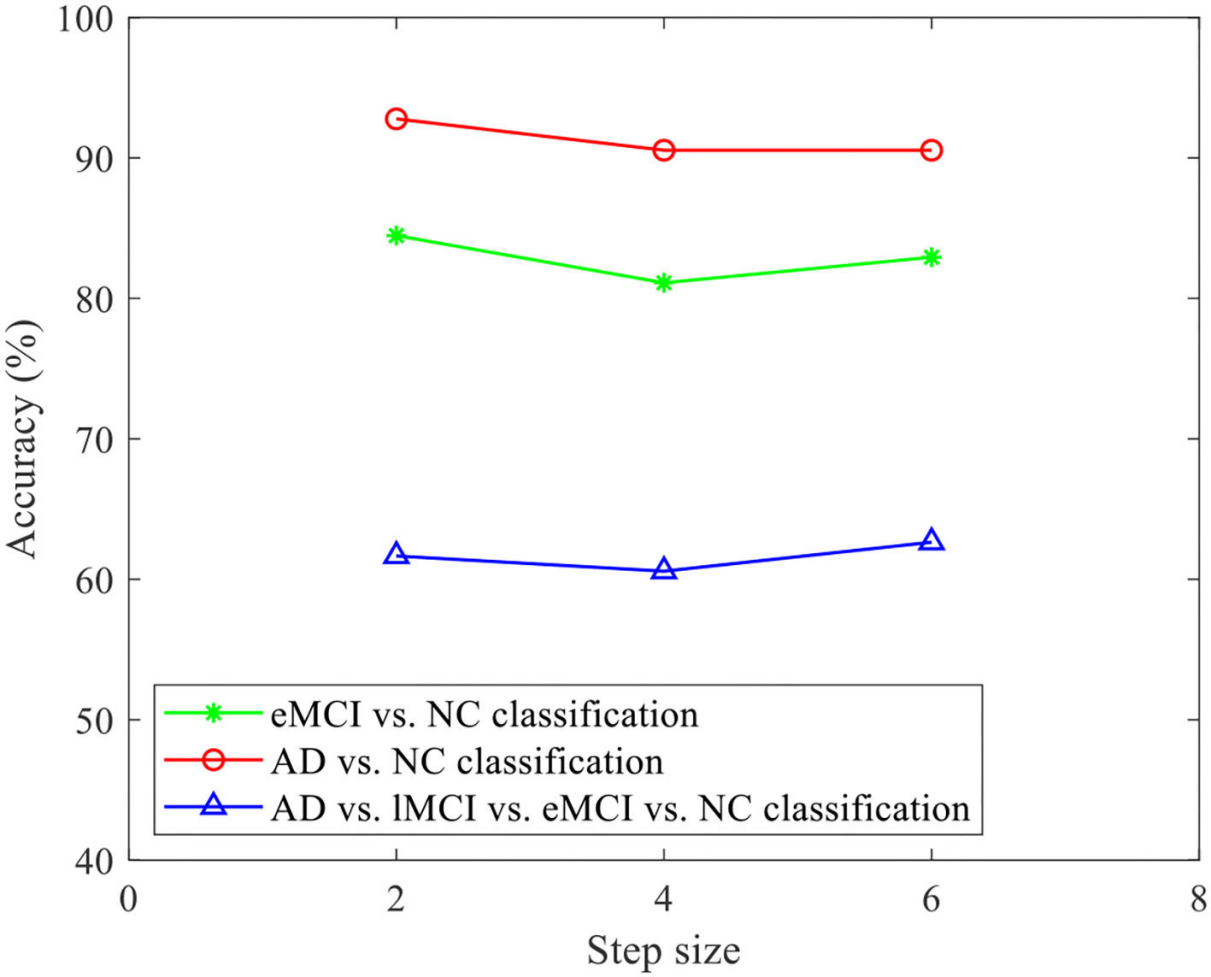
Accuracy of the proposed CRNN method with different stride sizes on three classification tasks (i.e., eMCI vs. NC, AD vs. NC, and AD vs. lMCI vs. eMCI vs. NC classifications).

Furthermore, in order to evaluate the effect of stride size on the classification of our proposed CRNN, we fix the sliding window length *L* = 70, and set the parameters *S* to 2, 4, and 6 for classification. Figure 10 illustrates the obtained accuracies of our CRNN method with three *S* values on three classification tasks. From Figure 10, the curve of the accuracy of our proposed CRNN method with different values of *S* is very smooth on the three classification tasks, indicating the robustness of our proposed CRNN method for different *S* values.

### 5.2. Temporal Dynamic Analysis

Previous studies have shown that rs-fMRI scan data is the synchronous fluctuation of the BOLD signal in the internal functional network of the entire brain (Lee et al., 2013), which has a high degree of temporal correlation. On the other hand, an LSTM with a chain of repeated neural network modules can analyze sequential information and learn long-term dependencies. From the perspective of model training, LSTM can effectively solve the problem of gradient explosion or gradient disappearance by using several gating units to control information flow. Based on the above reasons, we use LSTM to analyze the brain network, learn the sequential characteristics of the dynamic FC network, and use it for the classification of AD-related brain diseases.

From Figure 9 and Tables 2, 3, compared with CNN, our proposed CRNN method can obtain better classification performance. This also proves that learning sequential features through LSTM (as we do in CRNN) helps boost the classification performance. The possible reason could be that LSTM is able to model the underlying relationship between the time series of each subject and make decisions based on all time points instead of each single time point.

### 5.3. Limitations and Future Study

Several limitations need to be considered. *First*, we focus on using only rs-fMRI time series data to automatically identify AD/MCI in this study. In fact, different imaging methods (e.g., structural MRI and PET) can provide complementary information for disease diagnosis. The use of multi-modal information for brain network analysis will be our future work. *Second*, the construction of dynamic FC networks is independent of feature learning and classifier training, which may affect the prediction performance. It is interesting to integrate dynamic FC network construction, network feature learning, and classification into to unified framework. *Furthermore*, even though we used all rs-fMRI scans from all subjects of ADNI, the sample size in this work is still limited. In future study, we will evaluate the proposed method on a larger data set, such as attention deficit hyperactivity disorder (ADHD) with rs-fMRI data.

## 6. Conclusion

In this article, we developed a convolutional recurrent neural network (CRNN) for dynamic analysis of rs-fMRI time series data and automated diagnosis of AD-related brain diseases. In CRNN, we first construct dynamic functional connectivity (dFC) networks for each subject using an overlapping sliding time window strategy. These dFC networks are then fed into three convolutional layers for extracting the temporal features and an LSTM layer to capture the sequential information of temporal features along multiple time periods, followed by three fully connected layers for classification. Experimental results on 174 subjects with rs-fMRI from ADNI demonstrate the effectiveness of the proposed CRNN method in two binary classifications and one multi-class classification task.

## Data Availability Statement

Publicly available datasets were analyzed in this study. This data can be found here: Alzheimer's Disease Neuroimaging Initiative (ADNI) database (https://adni.loni.usc.edu).

## Ethics Statement

Ethical review and approval was not required for the study on human participants in accordance with the local legislation and institutional requirements. Written informed consent for participation was not required for this study in accordance with the national legislation and the institutional requirements.

## Author Contributions

KL, BJ, and PD contributed to the conception and design of the study. BJ organized the database. KL performed the experimental analysis and wrote the first draft of the manuscript. PD, XD, WB, and ML wrote sections of the manuscript. All authors contributed to manuscript revision, read, and approved the submitted version.

## Funding

KL, BJ, PD, XD, and WB were supported in part by NSFC (Nos. 61976006, 61573023, and 61902003), Anhui-NSFC (Nos. 1708085MF145 and 1808085MF171), and AHNU-FOYHE (No. gxyqZD2017010).

## Conflict of Interest

The authors declare that the research was conducted in the absence of any commercial or financial relationships that could be construed as a potential conflict of interest. The reviewer JH declared a past co-authorship with the authors BJ and ML to the handling editor.

## Publisher's Note

All claims expressed in this article are solely those of the authors and do not necessarily represent those of their affiliated organizations, or those of the publisher, the editors and the reviewers. Any product that may be evaluated in this article, or claim that may be made by its manufacturer, is not guaranteed or endorsed by the publisher.

## Footnotes

^1^ http://adni.loni.usc.edu/
