# Supplementary material for: Convolutional Recurrent Neural Network for Dynamic Functional MRI Analysis and Brain Disease Identification
Source: Front Neurosci. 2022 Jul 6;16:933660. doi: 10.3389/fnins.2022.933660 (PMC9298744; doi:10.3389/fnins.2022.933660)
Supplement: Supplementary file 1 [file Data_Sheet_1.PDF]

Supplementary Material

Table S1. Discriminative brain regions with significant difference of functional connectivity for eMCI vs. HC groups.

Name of ROI	Abbreviation
Precentral gyrus left	PrecentralL
Orbitofrontal cortex (superior) left	FrontalSupOrbL
Orbitofrontal cortex (middle) left	FrontalMidOrbL
Inferior frontal gyrus (opercular) left	FrontalInfOperL
Orbitofrontal cortex (inferior) left	FrontalInfOrbL
Rolandic operculum left	RolandicOperL
Olfactory left	OlfactoryL
Orbitofrontal cortex (medial) left	FrontalMidOrbL
Insula left	InsulaL
Anterior cingulate gyrus left	CingulumAntL
Middle cingulate gyrus left	CingulumMidL
Calcarine cortex left	CalcarineL
Cuneus left	CuneusL
Inferior occipital gyrus left	OccipitalInfL
Fusiform gyrus left	FusiformL
Supramarginal gyrus left	SupraMarginalL
Angular gyrus left	AngularL
Heshl gyrus left	HeschlL
Inferior temporal left	TemporalInfL
Left crus II of cerebellar hemisphere	CerebelumCrus2L
Left Lobule VI of cerebellar hemisphere	Cerebelum6L
Left lobule IX of cerebellar hemisphere	Cerebelum9L
Lobule IV, V of vermis	Vermis45
Lobule VII of vermis	Vermis7
Lobule IX of vermis	Vermis9

Table S2. Discriminative brain regions with significant difference of functional connectivity for AD vs. HC groups.

Name of ROI	Abbreviation
Precentral gyrus right	PrecentralR
Suplementary motor area right	SuppMotorAreaR
Olfactory right	OlfactoryR
Superior frontal gyrus (media) right	FrontalSupMedialR
Orbitofrontal cortex (medial) left	FrontalMidOrbL
Orbitofrontal cortex (medial) right	FrontalMidOrbR
Posterior cingulate gyrus right	CingulumPostR
ParaHippocampal gyrus left	ParaHippocampalL
Superior occipital gyrus right	OccipitalSupR
Middle occipital gyrus right	OccipitalMidR
Fusiform gyrus right	FusiformR
Postcentral gyrus right	PostcentralR
Superior parietal gyrus left	ParietalSupL
Superior parietal gyrus right	ParietalSupR
Supramarginal gyrus left	SupraMarginalL
Supramarginal gyrus right	SupraMarginalR
Caudate right	CaudateR
Heshl gyrus left	HeschlL
Superior temporal gyrus right	TemporalSupR
Temporal pole (superior) left	TemporalPoleSupL
Temporal pole (superior) right	TemporalPoleSupR
Left crus I of cerebellar hemisphere	CerebelumCrus1L
Right crus I of cerebellar hemisphere	CerebelumCrus1R
Right crus II of cerebellar hemisphere	CerebelumCrus2R
Left Lobule III of cerebellar hemisphere	Cerebelum3L
Right lobule IV, V of cerebellar hemisphere	Cerebelum45R
Right lobule VIII of cerebellar hemisphere	Cerebelum8R
Right lobule X of cerebellar hemisphere (flocculus)	Cerebelum10R
Lobule IX of vermis	Vermis9
